# Differential responses to kinase inhibition in FGFR2-addicted triple negative breast cancer cells: a quantitative phosphoproteomics study

**DOI:** 10.1038/s41598-020-64534-y

**Published:** 2020-05-14

**Authors:** Debbie L. Cunningham, Adil R. Sarhan, Andrew J. Creese, Katherine P. B. Larkins, Hongyan Zhao, Harriet R. Ferguson, Katie Brookes, Anna A. Marusiak, Helen J. Cooper, John K. Heath

**Affiliations:** 1 School of Biosciences, University of Birmingham, Edgbaston, Birmingham B15 2TT UK; 2 Department of Medical Laboratory Techniques, Nasiriyah Technical Institute, Southern Technical University, Nasiriyah, 6400 Iraq; 3 Immunocore, 101 Park Drive, Milton Park, Abingdon, Oxfordshire OX14 4RY UK; 4 Division of Molecular and Cellular Function, School of Biological Science, Faculty of Biology Medicine and Health, The University of Manchester, Manchester, M13 9PT UK; 5 Laboratory of Experimental Medicine, Centre of New Technologies, University of Warsaw, 02-097 Warszawa, Poland

**Keywords:** Breast cancer, Mass spectrometry

## Abstract

Fibroblast Growth Factor (FGF) dependent signalling is frequently activated in cancer by a variety of different mechanisms. However, the downstream signal transduction pathways involved are poorly characterised. Here a quantitative differential phosphoproteomics approach, SILAC, is applied to identify FGF-regulated phosphorylation events in two triple- negative breast tumour cell lines, MFM223 and SUM52, that exhibit amplified expression of FGF receptor 2 (FGFR2) and are dependent on continued FGFR2 signalling for cell viability. Comparative Gene Ontology proteome analysis revealed that SUM52 cells were enriched in proteins associated with cell metabolism and MFM223 cells enriched in proteins associated with cell adhesion and migration. FGFR2 inhibition by SU5402 impacts a significant fraction of the observed phosphoproteome of these cells. This study expands the known landscape of FGF signalling and identifies many new targets for functional investigation. FGF signalling pathways are found to be flexible in architecture as both shared, and divergent, responses to inhibition of FGFR2 kinase activity in the canonical RAF/MAPK/ERK/RSK and PI3K/AKT/PDK/mTOR/S6K pathways are identified. Inhibition of phosphorylation-dependent negative-feedback pathways is observed, defining mechanisms of intrinsic resistance to FGFR2 inhibition. These findings have implications for the therapeutic application of FGFR inhibitors as they identify both common and divergent responses in cells harbouring the same genetic lesion and pathways of drug resistance.

## Introduction

The Fibroblast Growth Factors (FGFs) are a family of evolutionarily conserved ligands that signal via binding to a cognate family of four structurally related transmembrane receptor tyrosine kinases, Fibroblast Growth Factor receptors 1–4 (FGFRs1–4)^1^. FGFR tyrosine kinase plays an essential role in many biological processes including regulation of development and differentiation, tissue regeneration, angiogenesis, cell migration, cell death and metabolism^2,3^. Genetic studies have shown that signalling via specific FGF ligands to different members of the FGFR family controls a wide variety of developmental events in embryonic, foetal, and postnatal development^4^. The intracellular pathways activated by FGFR signalling include the well-characterised RAS/RAF/MAPK/ERK and PI3K/PDK1/AKT pathways both of which involve sequential activation of protein (and lipid) kinases initiated by activated FGFR kinase mediated by phosphorylation of intermediate adaptor proteins such as FRS2, Grb2 and Gab1^5–7^. The biological functions of FGFs are, as a consequence, executed by the phosphorylation of the terminal targets of kinase-dependent pathways leading to specific changes in gene expression, cell motility, metabolism and survival.

Many forms of cancer involve manifestations of aberrant FGFR signalling^8^. The activity of FGF ligands regulates tumour/stromal cell interactions such as angiogenesis, metastasis and immune evasion^2,9^. It has become increasingly clear, from genomic sequencing studies, that mutation of FGFRs is a prominent feature of diverse common cancer types^10^. FGFR mutations in tumours exhibit diverse manifestations. Glioblastoma and haematological malignancies exhibit gene fusions leading to FGFR kinase activation via forced dimerisation of the kinase domain with partners such as TACC^11^. Many tumour types that exhibit point mutations in FGFR genes lead to ligand-independent, or hypersensitive signalling^12^. Amplification of FGFR gene loci are the most common lesions observed in specific tumour types such as breast (18% of tumours), endometrium and lung (13%) and ovary (9%)^13,14^. It is generally assumed that these mutations result in activation of FGFR kinase-dependent signalling pathways^15^ but the exact structure of these pathways and their terminal targets in tumour cells is poorly understood. Analysis of FGFR signalling in tumour cells harbouring mutant FGFRs is therefore required.

Given the prevalence of FGFR genetic lesions in human tumours, the pathway has become a target for drug development^16,17^. Different strategies have been employed to inhibit FGFR signalling the most common being the development of small molecule inhibitors of the FGFR kinase that interact with the ATP-binding cleft. These can exhibit multi-kinase specificity (e.g. Dovitinib^18^) or selective inhibition of the FGFR kinase (e.g. AZD4547^19^). Despite exhibiting potent inhibition of FGFR kinase activity in preclinical models, clinical trials with these agents have displayed weak efficacy^20^. Clinical responses are heterogeneous and vary according to the type of genetic lesion^21^. There is, for example, increasing evidence that small molecule inhibition is most effective in the cases of tumours harbouring FGFR amplifications and fusions but less in the case of point mutations^22^. An additional finding is the acquisition of drug resistance via second-site gatekeeper mutations in FGFRs^23,24^ or by re-activation of signalling via alternative receptor tyrosine kinases such HER2 and PDGFR-alpha^15,25^. It is also possible that activating mutations in critical elements of the FGFR signalling pathway such as PI3K/PTEN could lead to the acquisition of drug resistance^26^. These observations show that to improve the clinical efficacy of FGFR inhibitors more information is needed on the impact of these compounds on FGFR kinase-dependent signalling in the context of genetic lesions in the FGF pathway.

The biological consequences of genetic lesions in tumours are articulated through the protein networks in which the mutant gene products participate. These are far less well characterised, are often inferred rather than observed, and involve critical roles for non-mutant proteins which are not detected in mutational data. A central regulatory mechanism in these networks is post translational modification by protein phosphorylation of crucial regulatory sites that control protein activity, associations, localisation and abundance. This is the core mechanism that controls the biological behaviour of the tumour. Therefore, analysing patterns of protein abundance and phosphorylation gives direct access to the activity status of the pathway in the tumour; allows stratification based on pathway activity; and identifies key nodes for therapeutic targeting and novel nodes for drug development. In this study, we take a differential phosphoproteomics approach to analyse the impact of FGFR kinase inhibition on subsequent kinase-dependent phosphorylation events. Triple-negative breast tumours frequently harbour amplifications of FGFR1 or FGFR2^27^. FGFR-amplified cell lines can exhibit FGFR addiction^22^ in which cell proliferation and viability are critically dependent on continuous FGFR activity as inhibition by small molecule inhibitors leads to cell death. We selected two well-curated triple-negative breast tumour cell lines for analysis; SUM52^28^ and MFM223^29^. These exhibit high-level amplification of FGFR2, high levels of catalytically active FGFR2 protein and are addicted to FGFR2 activity as FGFR kinase inhibitors or RNAi-mediated silencing of FGFR2 induce cell death^22,30,31^. The design of the experiment is to stimulate the two cell lines with FGF in the presence or absence of the FGFR kinase inhibitor SU5402^32^ and analyse differences in phosphosite abundance within treatments and between the two cell lines using the differential labelling SILAC mass spectrometry technique^33^. This approach provides a comprehensive perspective on the structure of FGFR2 kinase-initiated signalling pathways; identifies phosphorylation events that are drug-dependent and reveals similarities and differences in responses between SUM52 and MFM223. The data also identifies potential biomarkers of drug response and mechanisms of intrinsic resistance to inform the design of future clinical trials^34,35^. The results reveal that, although sharing a common histopathological classification and FGFR2-addiction, the two cell lines differ significantly in their response to FGFR kinase inhibition.

## Results

### Proteomic landscapes of SUM52 and MFM223 cells

**Figure 1:**
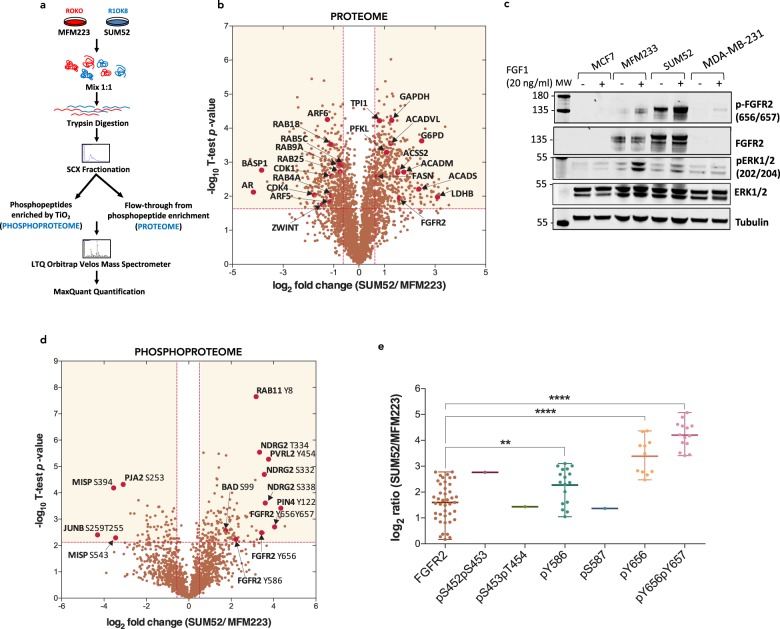
Differing proteomic and phosphoproteomic landscapes in two FGFR2-overexpressing triple negative breast cancer cell lines. (**a**) Schematic overview of experimental design. (**b**) Volcano plot showing the magnitude (log2 fold-change) and significance (−log10 Benjamini–Hochberg adjusted p-value) of differential protein abundance in SUM52 versus MFM223 cell lines. Proteins within the shaded areas have an abundance that is significantly different between the two cell lines (adjusted p value <0.05, >1.5 fold-change in abundance). (**c**) MCF7, MFM223, SUM52 and MDA-MB-231 cells were stimulated with 20 ng/mL FGF1 for 30 min. Levels of p-FGFR2 (pY656pY657), FGFR2, p-ERK (pT202pY204), ERK and tubulin in unstimulated and FGF1-stimulated whole cell lysates were analysed by western blotting. For uncropped images of blots see Supplementary Fig. S4. (**d**) Volcano plots showing the magnitude and significance of differential phosphopeptide abundance in SUM52 versus MFM223 cell lines. Phosphopeptides within the shaded areas have an abundance that is significantly different between the two cell lines (adjusted p value <0.05, >1.5 fold-change in abundance). (**e**) Differential abundance of FGFR2 peptides in SUM52 versus MFM223 cells. Each data point represents a single identification of a FGFR2 peptide (****P < 0.001, **P < 0.01).

Genetic alterations, such as receptor overexpression, can influence the activity and abundance of proteins within the host cell. Considering that these proteins act within the constraints of a unique, context specific network across different types of cells, knowledge of the proteomic landscape may help provide insights into predicted drug responses in different cell lines harbouring FGFR amplification. We used a SILAC quantification approach^33,36–39^ (Fig. 1a) to compare the proteomes of the FGFR2-overexpressing triple-negative breast cancer cell lines SUM52 and MFM223. In total, 80 peptide fractions were analysed, yielding 185,655 non-redundant and 29,920 redundant peptide identifications. These peptide sequences mapped to 3,659 proteins each with a minimum of two unique peptide identifications; 2,902 of which had associated quantitation data in at least three replicates, including two biological replicates (Supplementary Table S1). The abundance of 923 proteins was significantly different between SUM52 and MFM223 cells; 473 proteins had a high SUM52/MFM223 ratio, and 450 proteins had a low SUM52/MFM223 ratio (Fig. 1b**;** Supplementary Table S2).

Consistent with previous reports, the abundance of FGFR2 in SUM52 was higher than in MFM223 (Fig. 1b); the SUM52/MFM223 ratio was 3, similar to comparable mRNA levels^40^. Western blotting confirmed the differential abundance and substantial overexpression of FGFR2 in both cell lines compared to the non-amplified cell lines MCF7 and MDA-MB-231 (Fig. 1c). MFM223 are reported to overexpress the androgen receptor (AR)^29^: the abundance of the androgen receptor was 18-fold higher in MFM223 compared to SUM52 confirming this finding (Fig. 1b). Gene Ontology (GO) enrichment analysis was performed to further analyse the differences in the abundance of proteins between the two cell lines. Analysing proteins with a higher abundance in SUM52 revealed enrichment of proteins involved in metabolic processes (Supplementary Fig. S1). Cancer cells are well known to undergo metabolic reprogramming to support their growth and survival within a nutrient-poor environment. The abundance of several glycolytic enzymes was higher in SUM52 than MFM223 cells, including, phospho-fructose kinase (PFKL), triosephosphate isomerase (TPI1), glyceraldehyde 3-phosphate dehydrogenase (GAPDH), phosphoglycerate mutase (PGAM1), lactate dehydrogenase B (LDHB), and glucose-6-phosphate 1-dehydrogenase (G6PD) (Fig. 1b). These data suggest that SUM52 cells are more reliant on metabolic reprogramming for survival than MFM223 cells. Analysis of proteins with a higher abundance in MFM223 cells revealed an enrichment of proteins involved in cell-cell adhesion, protein transport, and actin-dependent processes (Supplementary Fig. S1). A group of GTPases involved in vesicular mediated transport of proteins: RAB4A, RAB5C, RAB9A, RAB18, RAB25, ARF5 and ARF6 are more abundant in MFM223 than in SUM52 (Fig. 1b). Increased expression of many of these is associated with increased cell motility, invasion of cancer cells and poor prognosis^41,42^.

Although these two triple-negative FGFR2-overexpressing cell lines both display an addiction to FGFR2 activation, it is apparent that the proteomic landscapes differ, which may impact on their global response to inhibitor treatment.

### Phosphoproteome analysis of SUM52 and MFM223

In addition to the differential proteome analysis, knowledge of the differential phosphoproteome can also provide valuable insight into the possible mechanisms used by the cancer cells to survive. Samples were prepared as shown in Fig. 1a. In total, 79 peptide fractions were analysed, yielding 1,929 unique phosphosites mapping to 1,130 unique proteins (Supplementary Table S3). The abundance of 549 phosphopeptides was significantly different between SUM52 and MFM223 cells; 396 had a high SUM52/MFM223 ratio, and 153 had a low SUM52/MFM223 ratio (Fig. 1d**;** Supplementary Table S4).

The FGFR2 activation loop tyrosines, Y656 and Y657, are autophosphorylation sites required to be phosphorylated for full activation of FGFR2 kinase activity^43–45^. It was evident that FGFR2 autophosphorylation on these sites was increased in SUM52, well beyond relative protein levels (Fig. 1e), suggesting that FGFR2 overexpression in SUM52 is associated with an increased ability to autophosphorylate when compared to MFM223. *In vitro* studies of the related FGFR1 kinase domain suggest an ordered pattern of phosphorylation events following domain dimerisation: Y466, Y586, Y588, Y656, Y657 and Y733 (using FGFR2 numbering). The singly phosphorylated Y656 peptide is also highly enriched in SUM52. However, mutation of the equivalent residue in FGFR1, Y653 to Phe has no impact on kinase activity^43–47^. Mutation of FGFR1 Y654 (equivalent to FGFR2 Y657) inhibits kinase activity and is thought to boost intrinsic kinase activity ~10-fold after initial phosphorylation of residues earlier in the activation sequence, including Y653. FGFR2_Y657 singly phosphorylated peptide was not detected suggesting the occurrence of phosphorylated Y657 is low compared to Y656 singly, or Y656Y657 doubly phosphorylated peptides. This is consistent with sequential phosphorylation seen in FGFR1, which requires Y653 to be phosphorylated before Y654 for maximal activation^45^.

One double phosphorylated peptide with a very low SUM52/MFM223 ratio is the JunB phosphopeptide T255S259. Both T255 and S259 on JunB are part of the phospho-degron motif recognised by the E3 ubiquitin ligase SCFFBXW7^48^. Ubiquitination of JunB leads to proteasomal degradation and down-regulation of JunB-regulated transcriptional activity. S259 is the priming phosphorylation site that initiates phosphorylation of T255 (and S251) by GSK3β. Under growth factor stimulated conditions, JunB levels increase due to downregulation of the phosphorylation of this phospho-degron, mediated by the inhibitory effects of Akt on GSK3β. The low SUM52/MFM223 ratio suggests that JunB is more stable in SUM52 than MFM223 cells.

A singly phosphorylated peptide with significantly higher abundance in SUM52 than MFM223 is PIN4_Y122. Signalling through PIN4_Y122 in glioblastoma cells with FGFR3-TACC gene fusion has been associated with tumour survival via regulation of mitochondrial metabolism^49^. Another phosphopeptide with significantly higher abundance in SUM52 than MFM223 is BAD_S99. Phosphorylation of serine 99 by AKT or p70S6K (RPS6KB1) inhibits apoptosis by preventing the pro-apoptotic interaction between BAD and anti-apoptotic BCL2 proteins^50^. This may indicate different mechanisms controlling apoptosis between the cell lines.

### Phosphoproteome sensitivity to FGFR kinase inhibitor treatment

**Figure 2:**
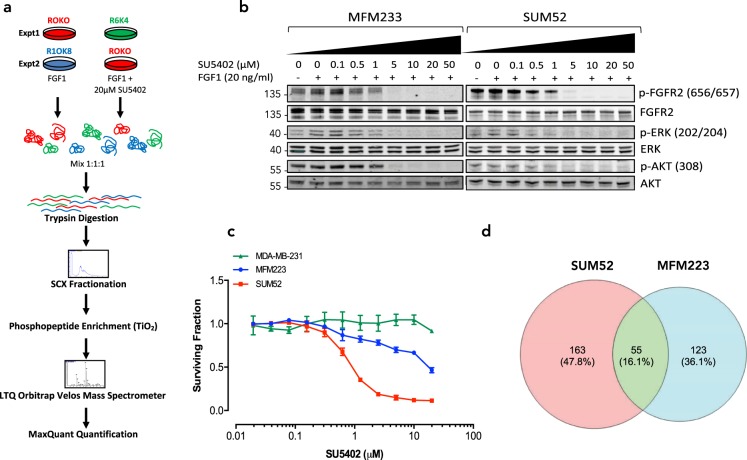
Inhibition of FGFR2 activity in SUM52 and MFM223 cells results in diverse changes in phosphorylation. (**a**) Schematic overview of experimental design. (**b**) MFM223 and SUM52 cells were stimulated with 20 ng/mL FGF1 for 30 min in the presence of increasing concentrations of SU5402. Levels of p-FGFR2 (pY656pY657), FGFR2, p-ERK (pT202pY204), ERK, p-AKT (pT308), and AKT in whole cell lysates were analysed by western blotting. For uncropped images of blots see Supplementary Fig. S5. (**c**) Cell viability was determined in the presence of increasing concentrations of SU5402. (**d**) Numbers of shared and diverse SU5402-sensitive phosphosites in SUM52 and MFM223 cells.

From a clinical perspective, patients harbouring triple-negative breast cancers that exhibit amplified FGFR2 are potential candidates for receiving treatment with an FGFR kinase inhibitor. However, the full extent of the downstream effects of this inhibition is unknown. In order to address this, we used SILAC-based quantitative phosphoproteomics to identify phosphorylation events that changed within the two FGFR2-overexpressing triple-negative breast cancer cell lines MFM223 and SUM52 upon treatment with the FGFR inhibitor SU5402^32^. Cells were either left untreated or pre-treated with SU5402 before stimulation with FGF1 for a further 30 min (Fig. 2a). The concentration of SU5402 used inhibited phosphorylation and activation of FGFR and downstream targets ERK and AKT (Fig. 2b), and resulted in >50% cell death in both cell lines after 72-hour treatment compared to >10% in MDA-MB-231 cells that do not overexpress the FGFR2 receptor (Fig. 2c). In total, 266 peptide fractions were analysed, yielding 6,574 unique, high-confidence phosphosites on 2,649 proteins (Supplementary Table S5). The distribution of phospho-amino acids identified was similar in both cell lines: MFM223 (82% serine, 15% threonine and 3% tyrosine); SUM52 (81% serine, 16% threonine and 3% tyrosine). Quantitation data was obtained for 3,082 phosphopeptides in SUM52 and 2,493 in MFM223, in at least two biological repeats. SU5402 treatment resulted in a significant decrease in abundance of 197 phosphopeptides in SUM52 (6.4%) and 157 (6.3%) in MFM223, and a significant increase in abundance of 21 phosphopeptides in SUM52 (0.7%) and 21 (0.8%) in MFM223 (Supplementary Table S6). If this is extrapolated to the 230,000 phosphosites estimated in the human proteome^51^, ~15,000 participate in the FGFR signalling pathway when defined by sensitivity to SU5402. The distribution of phospho-amino acids sensitive to SU5402 treatment differed between cell lines: MFM223 (85% serine, 10% threonine and 5% tyrosine); SUM52 (74% serine, 15% threonine and 11% tyrosine). There were large numbers of serine/threonine phosphorylation events sensitive to SU5402 in both, indicating that serine/threonine-directed kinases or phosphatases propagate downstream events of the tyrosine-directed FGFR2 kinase.

A total of 218 phosphosites were SU5402-sensitive in SUM52 and 178 in MFM223 with 55 (16%) exhibiting shared SU5402-sensitivity across both cell lines (Fig. 2d), highlighting a differential response to FGFR2 inhibition.

### Gene ontology and kinase enrichment analysis (KEA)

**Figure 3:**
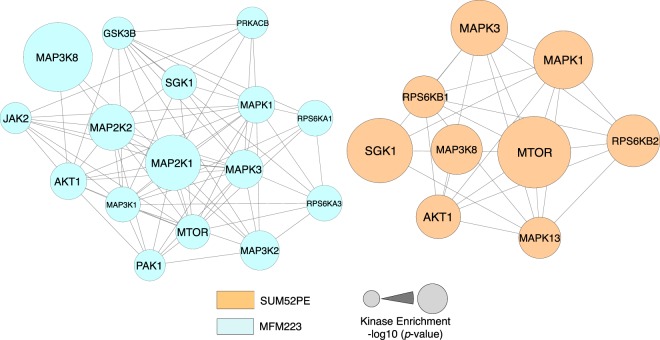
Distinct kinase nodes are predicted to be sensitive to FGFR2 inhibition. Phosphorylation sites sensitive to SU5402 treatment were searched using the Kinase Enrichment Analysis tool, KEA2. Each node represents an individual kinase and an edge connecting two nodes indicates that the corresponding kinase groups were predicted to phosphorylate at least one common residue. Node size corresponds to the kinase enrichment p-value.

Proteins containing SU5402-sensitive phosphosites were analysed for enrichment of Gene Ontology (GO) terms to gain insights into processes impacted by inhibition of FGFR2 activity. This analysis revealed different patterns of enrichment in the two cell lines (Supplementary Fig. S2). Next, we employed the Kinase Enrichment Analysis (KEA2) tool^52^ which tests a literature-curated database of kinase/substrate pairs against a submitted list of phosphosites to identify kinases that are predicted with high probability to be active in the test sample. The output of this analysis (Fig. 3**;** Supplementary Table S7) showed that the two cell lines were predicted to differ in their repertoire of active kinases. MFM223 scored most highly for MAP3K8 (COT) and MAP2K1 (MEK1), and SUM52 scored most highly for the MTOR and SGK1. The indication from this bioinformatics analysis is that the two cell lines exhibit differing repertoires of kinase activity as measured by the response to SU5402.

### FGFR Pathway analysis

**Figure 4:**
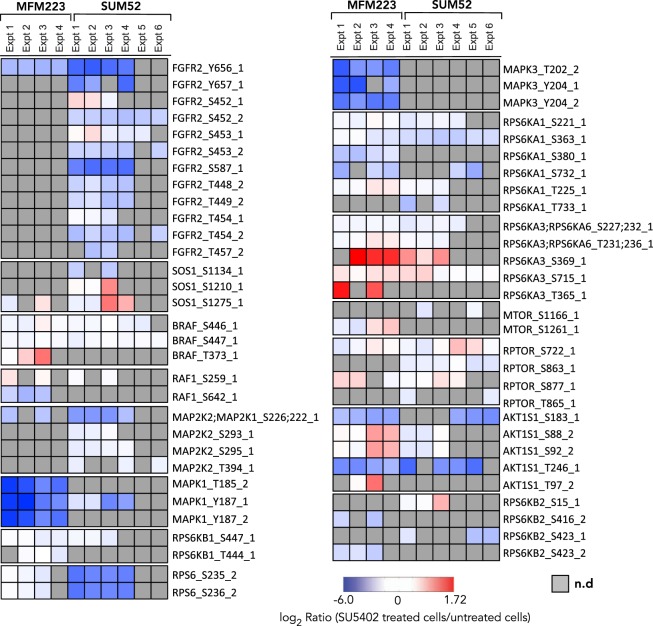
SU5402-sensitivity in proteins known to function downstream of FGFR2. Phosphopeptides belonging to proteins known to be regulated downstream of FGFR2 activation were extracted from the dataset. The ratios comparing cells in the absence or presence of SU5402 across all experimental repeats are plotted on a heatmap. _1, singly phosphorylated peptide; _2, doubly phosphorylated peptide.

Phosphosites from proteins previously identified as involved in FGFR signalling were extracted from the data to gain an understanding of FGFR pathway activity at the level of individual components (Fig. 4**;** Supplementary Fig. S3). Individual phospho-sites on these proteins were analysed using the iPTMnet data resource^53^ which curates kinase/substrate phosphorylation events assembled from the literature to which functional consequences can be ascribed, and phosphosites identified from large-scale proteomics screens whose function is unknown.

Phosphorylation of FGFR2_Y656 in the kinase activation loop was observed to be down-regulated in response to SU5402 treatment in both cell lines. FGFR2_Y657 and S587, also in the kinase activation loop, are inhibited by SU5402 in SUM52, but the corresponding peptides were not found in the MFM223 data. In SUM52 cells we also observed SU5402-mediated inhibition of Ser/Thr phosphorylation of FGFR2 in a cluster of acidic residues located N-terminal to the kinase domain: FGFR2_T448, T449, S452, S453, T454 and T457. The function of these sites is unknown but this is evidence for SU5402-sensitive feedback phosphorylation of FGFR2 via activation of downstream Ser/Thr kinases. The inhibitory effect was only observed on doubly phosphorylated peptides containing pT448pT339, pS452pS453, pT454pT457; the singly phosphorylated peptides containing pS452, pS453, or pT454 were not affected by the presence of SU5402.

Following activation of intrinsic FGFR kinase activity, part of the downstream signalling mechanism involves phosphorylation of the receptor-associated docking protein FRS2 and the recruitment of docking proteins such as SOS1, Grb2, SHP2 and Gab1^54–56^. Phosphorylation sites identified within the guanine nucleotide exchange factor SOS1, a Grb2 binding partner, were S1134 and S1210 in SUM52, and S1275 in both cell lines. Levels of SOS1_S1134 phosphorylation decreased in the presence of SU5402. SOS1_S1134 forms a binding site for 14-3-3 proteins when phosphorylated by the down-stream kinases RPS6KA1/3 (RSK1/2) leading to attenuation of signalling to the MAP Kinase pathway^57^. These data indicate that, in SUM52, the SOS1/Grb2 association is in an inhibited state which is released by the addition of SU5402, mediated by RSK signalling.

Active SOS1 regulates activity of the RAS-RAF-MAPK cascade by directly activating RAS which in turn recruits RAF leading to its subsequent activation. Phosphorylation of one RAF isoform, BRAF was detected in both cell lines. Phosphorylation of BRAF on S446 and S447 is insensitive to SU5402 treatment in both SUM52 and MFM223. S446 phosphorylation by an unidentified Ser/Thr kinase creates a negatively charged N-terminal region that elevates basal BRAF kinase activity and primes BRAF for binding RAS-GTP by regulating the interaction with RAS-GTP^58^. The additional negative charge on the adjacent residue S447 has also been shown to contribute to BRAF priming^58^. The autophosphorylation site BRAF_T373^59^ was also SU5402-insensitive, however, only detected in MFM223. Thus, BRAF exhibits basal kinase activity and priming that is not inhibited by SU5402.

Another RAF isoform, RAF1, is phosphorylated on S259 in both cell lines and not sensitive to SU5402 treatment. RAF1_S259 is phosphorylated by AKT, creating a 14-3-3 binding site, blocking association with RAS-GTP^60,61^ and inhibiting membrane association^62^. Phosphorylated RAF1_S259 is insensitive to growth factor stimulation^63^ and is de-phosphorylated by PP1–PP2A to activate MAPK^64^. Mutation of RAF1_S259 to inhibit its phosphorylation elevates basal kinase activity by enhancing membrane association and RAS-GTP binding leading to Noonan inhibition syndrome^65^. This suggests that RAF1 in both cell lines is in an, at least partially, inhibited cytosolic conformation. RAF1_S642 was also detected only in MFM223 cells and was found to be SU5402-sensitive. MAPK1 (ERK2) mediates phosphorylation of RAF1_S642, inhibits the RAS/RAF-1 interaction and desensitises RAF-1 to stimulation^66^. Thus, SU5402-sensitive ERK activation in MFM223 cells leads to relief of inhibition of RAF1 activity.

Activation of RAF activates the dual-specificity protein kinases MAP2K1/MEK1 and MAP2K2/MEK2 to propagate signalling to MAP-Kinases. The homologous phosphopeptide MAP2K1_S222/MAP2K2_S226 was observed to be inhibited by SU5402 in both SUM52 and MFM223. These residues are subject to basal phosphorylation by PDK1^67^ and induced phosphorylation via RAF1/BRAF and are essential for MAP2K1/MAP2K2 catalytic activity^68–70^. Thus, SU5402 mediates inhibition of MAP2K1/2 catalytic activity, despite the evidence that BRAF and RAF1 are in a partial or wholly inhibited activity state.

The ERKs MAPK1 (ERK2) and MAPK3 (ERK1) are the targets for phosphorylation by MAP2K1/2^71–73^. SU5402 treatment caused a decrease in the abundance of the doubly phosphorylated MAPK1_T185Y187 and the singly phosphorylated Y187 peptides in MFM223. Although the doubly phosphorylated peptide was not detected in SUM52, the abundance of the singly phosphorylated Y187 peptide was also decreased in the presence of SU5402. These residues are targets for phosphorylation by MAP2K1/2 and are required for full activation of MAPK kinase activity^74–76^. In the presence of SU5402, MAPK1 activity is therefore inhibited in both cell lines. Phosphorylation of the doubly phosphorylated MAPK3_T202Y204, both residues also targets for phosphorylation by MAP2K1/2 and required for full activation of MAPK kinase activity, was observed to be inhibited by SU5402 in MFM223. The singly phosphorylated MAPK3_Y204 was also detected and was SU5402-sensitive in MFM223 but not detected in SUM52 cells.

The ribosomal protein S6 kinases RPS6KA1(Rsk1) and RPS6KA3 (Rsk2) are downstream targets of the RAF/MEK/ERK pathway and mediate many cellular responses to ERK activation^77^ including negative feedback via phosphorylation of SOS. RPS6KA1_S221 was found to be constitutively phosphorylated in both SUM52 and MFM223 and unresponsive to SU5402. This site is constitutively phosphorylated by PDK1 at the plasma membrane and inducibly via activation of ERK activity^78,79^ and contributes to increased activity that also depends on phosphorylation of S363 and S380. RPS6KA1_S363 is an ERK substrate in the N-terminal kinase linker region^78,80^ and phosphorylation at this site is significantly reduced in SUM52 cells but not MFM223 in the presence of SU5402. RPS6KA1_S380 autophosphorylation is also inhibited by SU5402 in MFM223 cells but not detected in SUM52. Phosphorylation of S380 provides a docking site for phosphoinositide-dependent kinase 1 (PDK1)^81,82^ which can then phosphorylate S221 in the activation loop of the N-Terminal kinase domain. RPS6KA1_S380 autophosphorylation requires prior ERK-mediated phosphorylation of T573 which was not detected^83^. Thus, SU5402 supresses Rsk1 activity via inhibition of ERK activity in both cell lines.

Phosphorylation of RPS6KA3_S369 is elevated by SU5402 treatment in MFM223 but not SUM52 cells. RPS6KA3_T365 is also elevated in MFM223, but not detected in SUM52. RPS6KA3_S369 and RPS6KA3_T365 are reported ERK substrates^78,84^ required for activation of RSK2 kinase activity. However since MAPK1 and 3 are inhibited by SU5402, this may indicate regulation of these sites by alternative kinases. RPS6KA3_S227 (homologous to RPS6KA1_S221) is constitutively phosphorylated in both cell lines and uninhibited by SU5402 treatment. Thus, RPS6KA3 activity is induced by SU5402 in MFM223 cells but not SUM52.

An alternative pathway downstream of FGFR2 is the PI3K/AKT/mTOR/S6K pathway. Mammalian target of rapamycin (mTOR) is a conserved Ser/Thr kinase that forms two functionally distinct complexes via association with RPTOR (mTORC1) or RICTOR (mTORC2) important for nutrient and growth factor signalling^85^. mTOR activity is negatively regulated by association with AKT1S1(PRAS40). Constitutive SU5402-insensitive phosphorylation of mTOR phosphosite MTOR_S1261 was detected in MFM223 and MTOR_1166 in SUM52. Phosphorylation of MTOR_S1261 promotes phosphorylation of MTOR substrates and mTOR^86^. RPTOR is a mTOR adaptor protein that dictates mTORC1 complex substrate specificity. SU5402-insensitive phosphorylation of RPTOR_S722 and S877 was observed in both MFM223 and SUM52 and additionally RPTOR_S863 and RPTOR_T865 in SUM52 only. RPTOR_S863 and RPTOR_T865 are mTORC1-mediated rapamycin-sensitive phosphorylation sites and sensitive to MAPK and PI3K inhibition^87^. Phosphorylation of these residues primes RPTOR for subsequent phosphorylation of other sites including RPTOR_S877^88^. RPTOR_S722 is phosphorylated by AMPK^87,89^. Thus, RPTOR is in an SU5402-insensitive/mTORC1-activating configuration in both cell lines.

AKT1S1(PRAS40) binds the mTOR kinase domain and inhibits mTORC1 mediated signalling. Phosphorylation of AKT1S1_S183 and AKT1S1_T246 is inhibited by SU5402 treatment in both SUM52 and MFM223. AKT1S1_T246 is phosphorylated by AKT which leads to binding of 14-3-3 proteins and relief of AKTS1 inhibition^90,91^. Thus, treatment with SU5402 relieves a negative feedback pathway to mTORC1 kinase activity in both cell types. Taken together the evidence is that mTORC1 complex kinase is constitutively active and potentially further amplified by inhibition of FGFR kinase activity in both cell lines.

The S6 kinases RPS6KB1 (S6K1) and RPS6KB2 (S6K2) are downstream targets of mTOR^92^ that regulate cell responses such as apoptosis, metabolism and biosynthesis including regulation of ribosomal protein S6 phosphorylation^93^. RPS6KB1_S447 was observed to be uninhibited by SU5402 in both cell lines. Phosphorylation of this site is a rapamycin and wortmannin-sensitive priming step required for full activation of S6K1 kinase activity^94–96^. SU5402 inhibits RPS6KB2_S423 phosphorylation in both cell lines. Although no function has been described for this site, the sequence context is identical to RPS6KB1_S447 suggesting that S6K2 activity is inhibited in both cell lines. The S6K1 substrate residues in RPS6: RPS6_S235, S236^93,97,98^ are also inhibited by SU5402 in SUM52 but not MFM223.

### Differential phosphorylation of shared phosphosites

**Figure 5:**
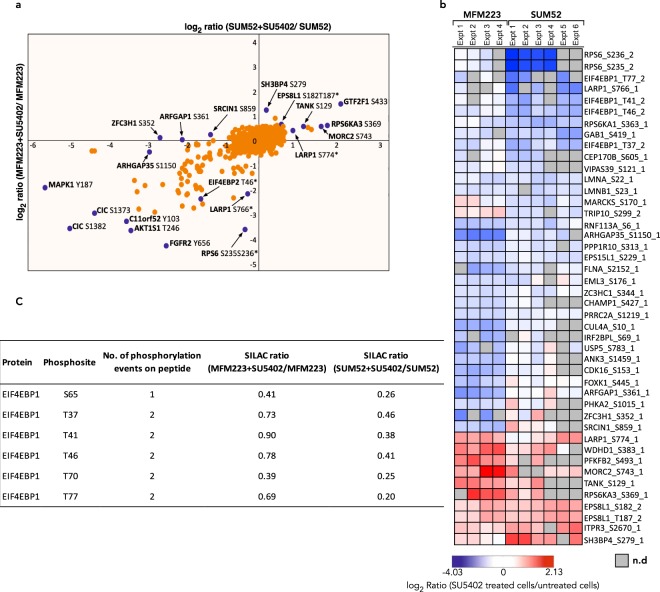
Differential regulation of SU5402-sensitive phosphosites. (**a**) SU5402-sensitive phosphosites identified in both cell lines are plotted. *, phosphosites identified on doubly phosphorylated peptides. (**b**) Heatmap illustrating the differences in ratios observed in phosphosites identified in both cell lines exhibiting differential SU5402-sensitivity. _1, singly phosphorylated peptide; _2, doubly phosphorylated peptide. (**c**) Differential SU5402-sensitivity of EIF4BP1 phosphopeptides was observed. The table lists median ratios for each phosphopeptide.

A comprehensive comparison of SU5402-inhibited phosphorylation events in the two cell lines using the pathway reconstruction approach above is limited by instances where phosphosites are only found in one of the two datasets. As an alternative approach, we created a database of the 1,385 phosphosites that were identified in both cell lines and examined divergence in SILAC ratios between the two cell lines (Fig. 5a). 52 phosphosites (4%) were down-regulated in both cell lines, 19 sites (1.5%) were down-regulated in MFM223 but not SUM52 and 15 (1%) down-regulated in SUM52 but not MFM223 (Fig. 5b). 1 phosphosite (0.1%) was up-regulated in both cell lines, 6 sites (0.4%) were up-regulated in MFM223 but not SUM52 and 4 (0.3%) up-regulated in SUM52 but not MFM223. Thus, there are phosphosites that exhibit different responses to SU5402 inhibition in the two cell lines.

A striking example of a differential response to SU5402 inhibition is Eukaryotic translation initiation factor 4E-binding protein 1 (EIF4EBP1) (Fig. 5c). EIF4EBP1 is a well-characterised mTOR substrate^99^. Phosphorylation of EIF4EBP1 at multiple sites induces release from EIF4E permitting cap-dependent protein translation. A singly phosphorylated peptide, EIF4EBP1_S65, and a doubly phosphorylated peptide containing phosphorylated T70 were inhibited by SU5402 in both cell lines whereas doubly phosphorylated peptides containing a phosphorylation at residues T37, T41, T46 or T77 were down-regulated in SUM52 but not in MFM223. EIF4EBP1_S65 and T70 are serum-sensitive and rapamycin-sensitive mTOR mediated phosphorylation sites^100,101^ but have also been reported to be sensitive to inhibition of GSK3β^102,103^ and MAPK^104^ in different settings. EIF4EBP1_T37 and T46 have also been identified as mTOR substrates^105,106^ and have been defined as priming sites whose phosphorylation is required for phosphorylation of EIF4EBP1_S65 and 70 and full inhibition of EIF4EBP1 binding to EIF4E^106^. We conclude that SU5402-mediated inhibition of EIF4EBP1_T37 and T46 occurs via inhibition of mTOR in SUM52 cells and EIF4EBP1_S65 and 70 via inhibition of MAPKinase (or an undefined kinase) in both cell lines. Downstream of mTOR, S6K1 substrate residues in RPS6: RPS6_S235 and S236^93,97,98^ are also inhibited by SU5402 in SUM52 but not MFM223.

As previously discussed, downstream of the RAS/MEK/MAPK pathway are the RSK kinases. RSK1 (RPS6KA1) activity is downregulated in SUM52 on addition of SU5402 and unchanged in MFM223, as evidenced by a decrease in S363 phosphorylation. In contrast, RSK2 (RPS6KA3) is upregulated on addition of SU5402 and unchanged in SUM52. These data show therefore that SU5402 differentially alters specific mTOR-mediated signaling and RAS/MEK/ERK events in SUM52 and MFM223. Thus, in the two cell lines, inhibition of FGFR kinase activity is propagated intracellularly to distinct functional outcomes.

We examined the common and divergent datasets for potential explanations for the FGFR2-addicted phenotype of SUM52 and MFM223 and induction of apoptosis in response to SU5402. MCL1_T163 is inhibited by SU5402 in SUM52. This is a substrate of MAPK^107^ and is required to inhibit degradation of anti-apoptotic MCL1. Thus, inhibition of MCL1_T163 phosphorylation will lead to depletion of MCL1 and execution of apoptosis. Phosphorylation of BAD_S99 is inhibited in both cell lines. Phosphorylation of BAD_S99 by AKT or RAF^108,109^ leads to the binding of 14-3-3 proteins and inhibition of pro-apoptotic function^110,111^. Thus, SU5402-mediated apoptosis involves simultaneous activation of pro-apoptotic and inhibition of anti-apoptotic functions.

## Discussion

This study set out to define the proteomic landscape and phosphorylation-dependent intracellular signalling pathways impacted by pharmacological inhibition of the FGFR2 kinase. It focuses on two triple-negative breast tumour cell lines, MFM223 and SUM52 that exhibit the shared property of amplification of the FGFR2 gene and protein and ongoing dependance on FGFR2 signalling for cell survival. The motivation is to better comprehend the molecular responses to FGFR2 activation in cancer and identify new biomarkers and targets for more effective use of agents directed at inhibition of FGFR2 signalling. Using a differential phosphoproteomics approach, we identify 341 sites on 219 proteins that are sensitive to inhibition of FGFR2 kinase activity. This data significantly expands the known landscape of FGFR2 signalling^7^ and identifies many new targets for future investigation. Using a pathway reconstruction approach, we observe inhibition of the canonical RAF/MEK/ERK/RSK, PI3K/AKT/PDK/mTOR/S6K pathways. By focusing on phosphorylation sites identified in both cell lines, we also observe significant differences in response to FGFR2 inhibition in the two cell lines. These are not, however, mutually exclusive as elements of both pathways are inhibited in both cell lines. Our conclusion is that the FGF pathway is a dynamic system whose behaviour is defined by quantitative and qualitative features of pathway architecture.

An essential issue in the clinical application of kinase-directed inhibitors is intrinsic resistance: the propensity of downstream responses to deploy compensatory changes to inhibit the pathway. These might be particularly important in the case of oncogene addiction mechanisms^112^ as, by definition, prolonged activation of a particular pathway might involve desensitisation before drug inhibition. Identification of intrinsic and induced resistance mechanisms is therefore crucial for stratifying tumours and understanding the heterogeneity of drug responses observed in clinical trials^20^. A prominent mechanism of intrinsic resistance revealed in this dataset is the use of Ser/Thr directed phosphorylation of binding sites for 14-3-3 proteins^113,114^. This can have both inhibitory and activating functions. AKT phosphorylates both BRAF and RAF1 at critical 14-3-3 binding residues which, in the 14-3-3 bound form, acts to inhibit membrane binding and the interaction with RAS-GTP thereby attenuating BRAF/RAF1 signalling^60,61,115^. This connects prior activation of the AKT pathway and suppression of the RAS/RAF/MEK/ERK pathway. The adaptor protein SOS1 is phosphorylated by MAPK and Rsk eliciting 14-3-3 binding which inhibits the association of Grb2 and signalling to RAS/RAF^57,116^. Both these pathways were observed to be insensitive to SU5402 indicating prior adaptation to amplified FGFR2. An example of induced resistance mediated by 14-3-3 proteins is the SU5402-senstive inhibition of phosphorylation of the mTOR complex inhibitor AKT1S1^90,91^. In this case the inhibtion of phosphorylation leads to release of 14-3-3 proteins, inducing binding of AKT1S1 to mTOR and inhibition of function.

Induced resistance can occur by the phosphorylation of upstream pathway elements by downstream kinases to dynamically mediate feedback inhibition. We observed SU5402-sensitive phosphorylation of RAF1 by MAPKinase which inhibits the RAS-GTP/RAF1 interaction^66^. We also observed SU5402-sensitive RAF mediated phosphorylation of MAP2K1 which acts to inhibit MAP2K1 activity^68,69^. The evidence from this data is that there exist multiple pathways regulating resistance to drug inhibition operating over different timescales. Both mechanisms provide potential explanations for heterogeneity of tumour responses to drug inhibition^20,22^.

SUM52 and MFM223 have previously been screened with panels of candidate therapeutic compounds to establish drug sensitivity profiles^26,117,118^. These data reveal differences in sensitivity to inhibition of specific pathways. SUM52 cells exhibit particular sensitivity to inhibitors of the mTOR pathway: Rapamycin, GSK2119563, GSK2126458, Temsirolimus^26^. MFM223 is insensitive to mTOR inhibitors, such as rapamycin, but exhibits sensitivity to inhibitors of the PI3K/AKT pathway such as Wortmannin, Akt Inhibitor IV and MK-2206^26,118^. Functional profiling of 714 kinases in siRNA screens^119,120^ showed that MFM223 and SUM52 are both sensitive to depletion of FGFR2 and MAPK3. MFM223 are also sensitive to depletion of PI3K, AKT3 and MAPK1. Both cell lines are resistant to depletion of PDK, AKT1/2 MAP2K1, MAP2K2, BRAF, RAF1, RPS6KA1/3, and RPS6KB1/2^119,120^. MFM223 has an activating mutation H1047R in PI3KCA^121,122^ whereas SUM52 are WT PI3KCA and PTEN deleted^27^. This evidence, taken with the phosphoproteomics data presented here, shows that the divergence in response to FGFR2 inhibition initiates in processes proximal to FGFR2 activation where signalling into the RAS/RAF/MEK/ERK/RSK and AKT/PDK/mTOR/S6K systems are regulated. Our results show that, in MFM223, FGFR2 kinase dependent signalling into RAS/RAF/MEK/ERK predominates whilst signalling into PI3K/AKT/PDK/mTOR/S6K is more prominent in SUM52 as previously suggested^22^. This suggests that deletion of PTEN and activating mutations of PI3K, and feedback processes controlling their activity, may have different quantitative effects on downstream pathways^123^.

Two aspects of this study have implications for improving the application of current therapies targeting FGFRs. High-level amplification and FGFR-addiction may lead to long-term desensitisation of downstream pathways and consequential blunting of the effects of drug inhibition. It is also conceivable that inhibiting FGFR signalling as a therapeutic strategy could lead to an increase in basal signalling by relief of inhibition. Second, not all FGFR-addicted cells exhibit the same response to pathway inhibition. This may involve, not only the consequences of additional mutations that impact pathway behaviour^13^ but quantitative differences in the abundance of pathway components^124^. This points to a pressing need to categorise tumours not only by their repertoire of somatic mutations but by analysis of pathway status before and after drug treatment. The data in this study, therefore, provides a catalogue of functional phosphoprotein biomarkers for further analysis and tumour stratification^34,35^.

## Methods

### Cell culture

SUM52 and MFM223 cells were cultured at 37 °C, 5% CO_2_ in RPMI-1640 or DMEM containing 2 mM L-Glutamine (Lonza), respectively, supplemented with 0.1 mg/ml streptomycin, 100 U/ml penicillin (Sigma-Aldrich), and 10% v/v fetal calf serum (Biosera). For SILAC labelling, SUM52 cells were prepared as previously described^125^ and MFM223 cells were cultured in SILAC DMEM (Thermo Fisher Scientific) supplemented with 0.798 mM L-lysine and 0.398 mM L-arginine (either isotopically “light” L-lysine and L-arginine (Sigma-Aldrich), “medium” 4,4,5,5-D4 L-lysine and ^13^C_6_ L-arginine, or “heavy” ^13^C_6_ L-lysine and ^13^C_6_^15^N_4_ L-arginine (CK Isotopes)), supplemented with 10% dialyzed FBS (Biosera), 0.1 mg/ml streptomycin, and 100 U/ml penicillin at 37 °C with 5% CO_2_.

### Cell treatment and cell lysis

Following 4 hrs serum starvation in serum-free media, cells were pre-treated with 20 μM SU5402 for 30 min, followed by addition of 20 ng/ml FGF1 and 20 mg/ml heparin for 30 mins, or treated as above in the absence of chemical inhibitor. Cell lysis and protein concentration determination were carried out as previously described^125^.

### Western blotting

Western blotting was carried out as previously described^36^. Primary antibodies used in this study were obtained from Santa Cruz Biotechnology (ERK, ERK pT202pY204), Cell Signaling Technology (FGFR1 pY653/pY654 (which also recognises FGFR2 pY656/657), AKT, and AKT pT308) and Sigma-Aldrich (tubulin).

### Cell viability assay

Cell viability was determined using the CellTiter-Glo assay (Promega). Cells were seeded at 7 ×10^3^ cells per well into opaque-walled, flat-bottom 96-well plates, and incubated overnight at 37 °C, 5% CO_2_. The medium was changed to the experimental conditions after 24 hours and incubated for 72 hours at 37 °C, 5% CO2. 100 μl of CellTiter-Glo Reagent was added into each well, incubated for 10 minutes at room temperature before measuring luminescence in a Tecan F200pro luminometer.

## Trypsin digestion, sample fractionation and phosphopeptide enrichment of samples

10 mg of each labelled cell population were pooled prior to trypsin digestion. Proteins were reduced with 8 mM DTT, alkylated with 20 mM iodoacetamide in 50 mM ammonium bicarbonate and digested with Trypsin Gold (Promega; 1:100 enzyme:protein ratio) at 37 °C overnight. Digested samples were acidified by addition of 0.5% TFA. Peptides were desalted using Sep-Pak C18 Cartridges (Waters) according to manufacturer’s instructions. Desalted and dried peptides were resuspended in 100 μL mobile phase A (10 mM KH_3_PO_4_, 20% acetonitrile, pH 3) and loaded onto a 100 × 4.6 mm polysulfoethyl A column (5 μm particle size, 200 nm pore size, PolyLC). Separation used a gradient elution profile that started with 100% mobile phase A, increased from 0 to 50% mobile phase B (10 mM KH_3_PO_4_, 20% acetonitrile, 500 mM KCl, pH 3) over 30 min, increased to 100% B over 5 min, and then returned to 100% A. The peptides eluted from the column were split into 20 fractions. Each fraction was desalted using a C8 macrotrap cartridge (Michrom Bioresources) according to manufacturer’s instructions. Phosphopeptides were enriched using TiO_2_ tips (Titansphere Phos-TiO kit, GL Sciences) as previously described^38^. Each phospho-enriched sample was split into two in order to run technical repeats.

### Mass spectrometry

On-line liquid chromatography was performed as previously described^38^. Peptides eluted directly (350 nL/min) via a Triversa nanospray source (Advion Biosciences) into a LTQ Orbitrap Velos mass spectrometer (Thermo Fisher Scientific). The mass spectrometer alternated between a full FT-MS scan (m/z 380–1600) and subsequent CID MS/MS scans of the seven most abundant ions. Survey scans were acquired in the Orbitrap cell with a resolution of 60,000 at m/z 400. Precursor ions were isolated and subjected to CID in the linear ion trap. Isolation width was 2 Th. Only multiply-charged precursor ions were selected for MS/MS. CID was performed with helium gas at a normalized collision energy of 35%. Precursor ions were activated for 10 ms. Data acquisition was controlled by Xcalibur 2.1 software.

### Identification and quantification of peptide and proteins

Mass spectra were processed using the MaxQuant software (version 1.5.3.8)^126,127^. Analysis of the comparative proteome and phosphoproteome between MFM223 and SUM52 cells included data from two biological replicates, each split into two technical replicates. Analysis of the SU5402-sensitive phosphoproteome included data from two biological replicates, each split into two technical replicates, plus data from a further biological replicate for SUM52 acquired as previously described^125^. Data were searched, using the Andromeda search engine within MaxQuant^128^ against the human Swiss-Prot database (downloaded 16.6.17). The human database contained 20,205 reviewed protein entries. The search parameters were: minimum peptide length 7, peptide tolerance 20 ppm (first search) and 4.5 ppm (main search), mass tolerance 0.5 Da, cleavage enzyme trypsin/P, and 2 missed cleavages were allowed. Carbamidomethyl (C) was set as a fixed modification. Oxidation (M), acetylation (Protein N-term), and phospho (STY) were set as variable modifications. The appropriate SILAC labels were selected and the maximum labelled amino acids was set to 3. All experiments were filtered to have a peptide and protein false-discovery rate (FDR) below 1% and the match between runs featured was enabled. Within the MaxQuant output, phosphorylation sites were considered to be localised correctly if the localisation score (PTM score) was at least 0.75. Bioinformatics analysis was performed using Perseus^129^. Significance testing was carried out using a Student’s *t*-test on log2 transformed ratios and controlled with a Benjamini-Hochberg FDR threshold of 0.05. Peptides quantified in three or more experimental repeats, including at least two biological repeats, were deemed significantly changed if they had a p-value of <0.05 and a ratio of <0.667 or >1.5 (at least a 1.5-fold change in abundance). Also deemed significantly changed were peptides identified in at least two biological repeats with a median ratio <0.5 or >2.0 with each individual ratio showing >1.5-fold change in the same direction.

### GO analysis and kinase motif analysis

DAVID (Database for Annotation, Visualization and Integrated Discovery)^130^ was used to identify over-represented GO terms within the datasets. Phosphopeptides containing well localised phosphosites were analysed for predicted kinase motifs using KEA^52^ with a high stringency setting.

## Supplementary information

Supplementary Figures

Supplementary Table S1

Supplementary Table S2

Supplementary .Table S3

Supplementary Table S4 

Supplementary Table S5 

Supplementary Table S6 

Supplementary Table S7 

## Data Availability

The mass spectrometry proteomics data, including the MaxQuant output, have been deposited to the ProteomeXchange Consortium via the PRIDE partner repository with the dataset identifier PXD016777^131^.
