# Supplementary material for: Differential responses to kinase inhibition in FGFR2-addicted triple negative breast cancer cells: a quantitative phosphoproteomics study
Source: Sci Rep. 2020 May 14;10:7950. doi: 10.1038/s41598-020-64534-y (PMC7224374; doi:10.1038/s41598-020-64534-y)
Supplement: Supplementary file 1 — Supplementary Figures [file 41598_2020_64534_MOESM1_ESM.pdf]

Differential responses to kinase inhibition in FGFR2-addicted triple negative breast cancer cells: a quantitative phosphoproteomics study

Debbie L. Cunningham[1*], Adil R. Sarhan[1,2], Andrew J. Creese[1,3], Katherine P.B. Larkins[1], Hongyan Zhao[1], Harriet R. Ferguson[1,4], Katie Brookes[1], Anna A. Marusiak[1,5], Helen J. Cooper[1], John K. Heath[1*]

[1]School of Biosciences, University of Birmingham, Edgbaston, Birmingham B15 2TT, UK

[2]Department of Medical Laboratory Techniques, Nasiriyah Technical Institute, Southern Technical University, Nasiriyah 6400, Iraq

[3] Immunocore, 101 Park Drive, Milton Park, Abingdon, Oxfordshire OX14 4RY, UK

[4]Division of Molecular and Cellular Function, School of Biological Sciences, FBMH, The University of Manchester, Manchester, UK

[5] Laboratory of Experimental Medicine, Centre of New Technologies, University of Warsaw, 02-097 Warszawa, Poland

*Corresponding authors

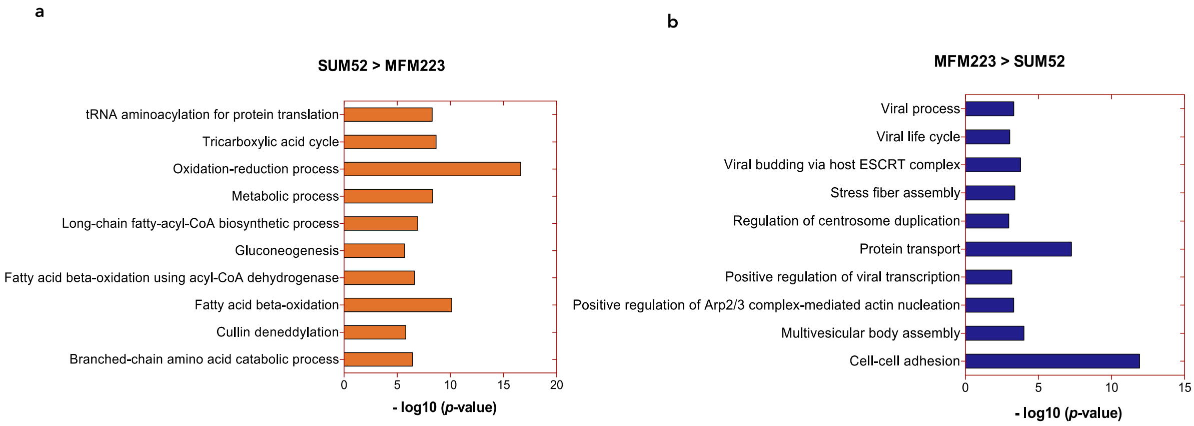

Supplementary Figure S1. Differentially expressed proteins were analysed in DAVID[1] to identify enriched GO Biological Processes. The top 10 enriched categories for proteins with a high SUM52/MFM223 ratio (a) or a low SUM52/MFM223 ratio (b) are plotted as bar charts.

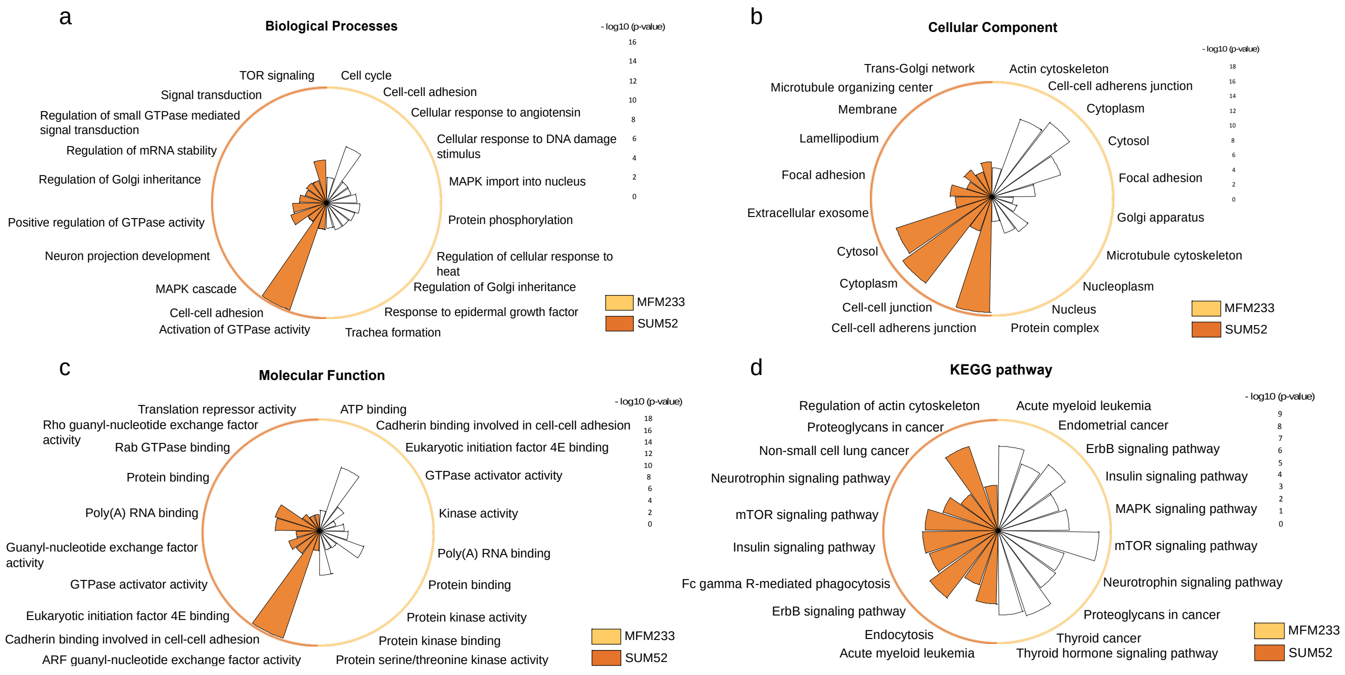

Supplementary Figure S2. GO-term analysis of differentially SU5402-sensitive phosphopeptides in SUM52 and MFM223 cells. Proteins which contained SU5402-sensitive phosphopeptides in either SUM52 or MFM223 cells were analysed in DAVID[1] to identify enriched GO Biological Processes (a), Cellular Components (b), Molecular Functions (c) or KEGG pathways (d). The top 10 enriched categories are plotted within a radar chart.

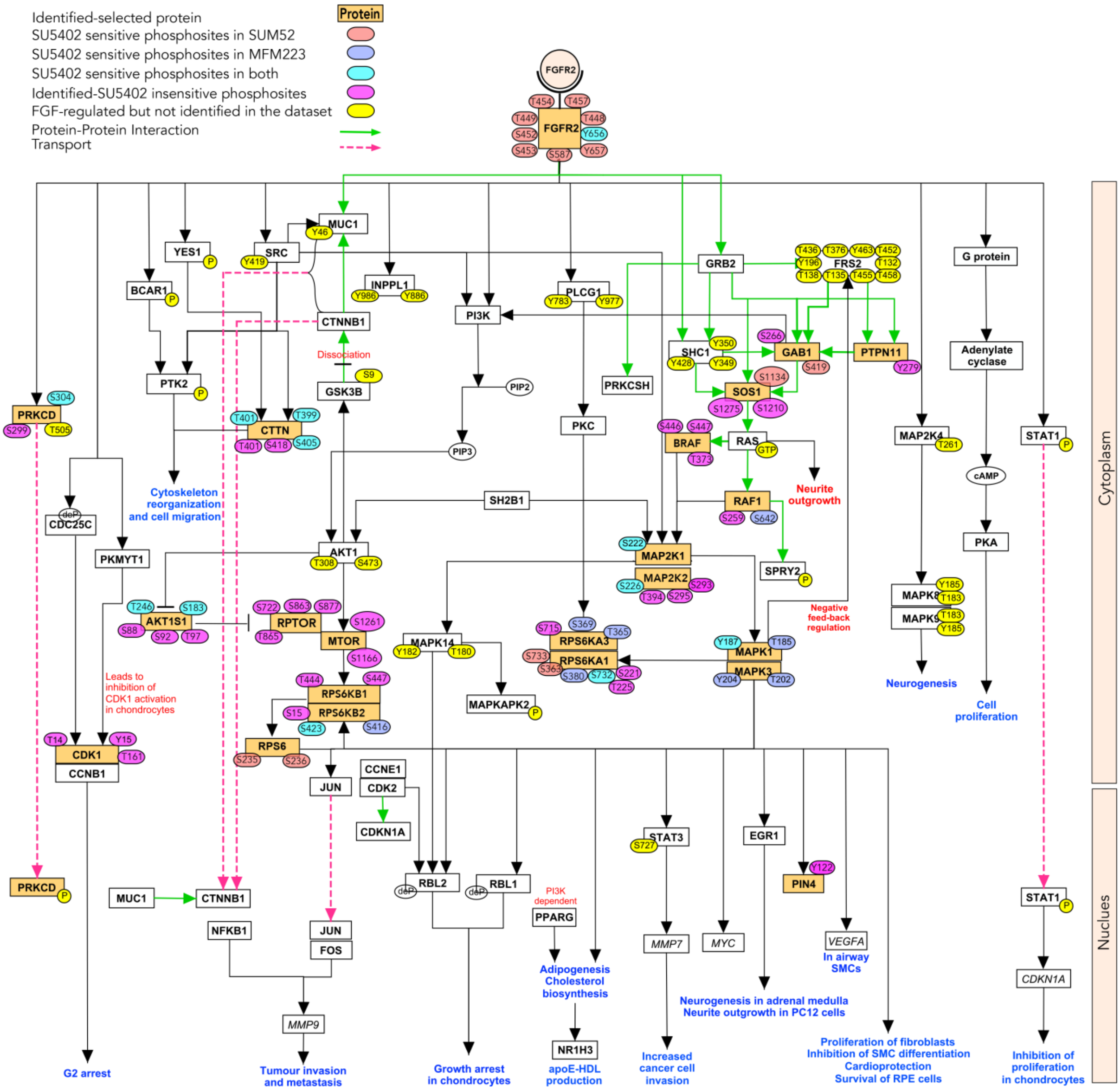

Supplementary Figure S3. A detailed map of FGFR2 signalling created by NetPath[2]. SU5402 sensitive and insensitive phosphosites found within MFM223 and SUM52 dataset visualised within the FGF signalling network**.**

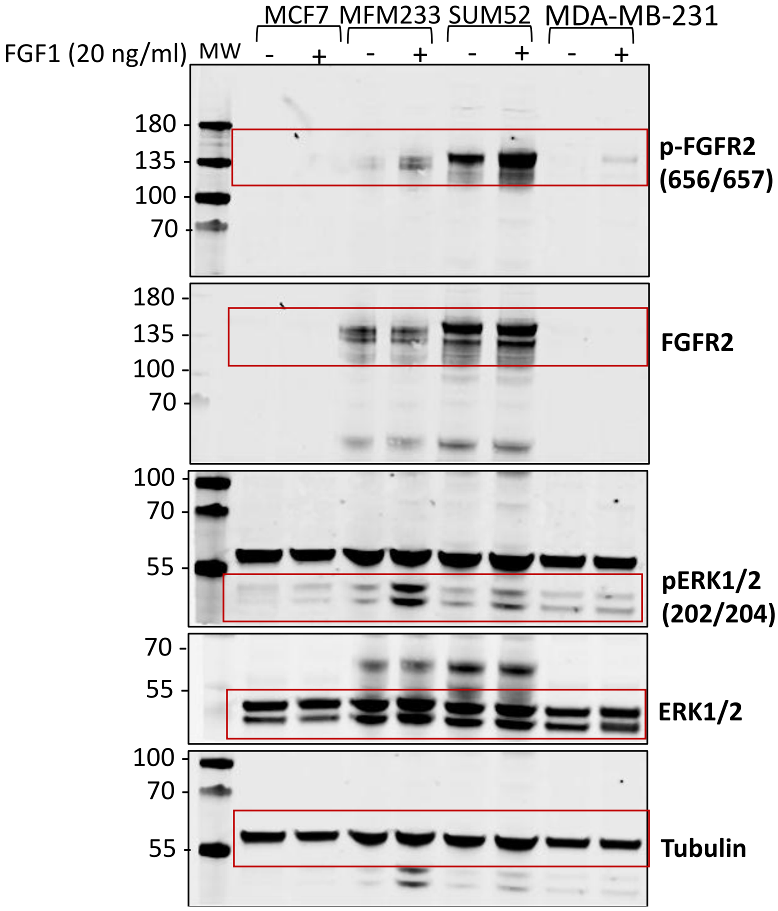

Supplementary Figure S4. MCF7, MFM223, and SUM52 cells were stimulated with 20 ng/mL FGF1 for 30 min. Levels of p-FGFR2 (pY656pY657), FGFR2, and tubulin in unstimulated and FGF1-stimulated whole cell lysates were analysed by western blotting. Boxes indicate cropped regions displayed in Figure 1c.

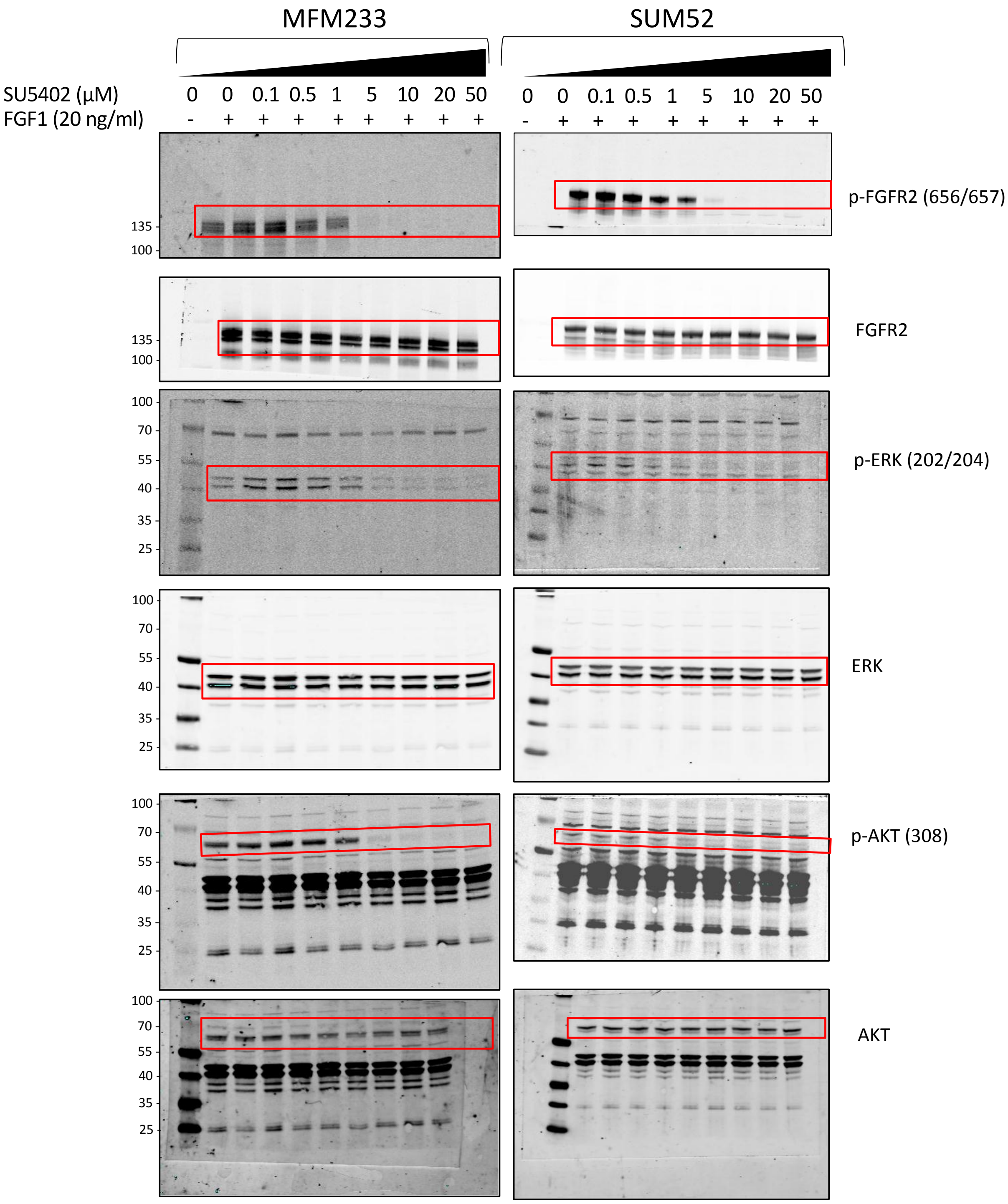

Supplementary Figure S5. MFM223 and SUM52 cells were stimulated with 20 ng/mL FGF1 for 30 min in the presence of increasing concentrations of SU5402. Levels of p-FGFR2 (pY656pY657), FGFR2, p-ERK (T202Y204), ERK, p-AKT (T308), and AKT in whole cell lysates were analysed by western blotting. Boxes indicate cropped regions displayed in Figure 2b.

Supplementary References

1. Huang, D. W., Sherman, B. T. & Lempicki, R. A. Systematic and integrative analysis of large gene lists using DAVID bioinformatics resources. *Nat. Protoc.* **4**, 44–57 (2009).

2. Raju, R. et al. NetSlim: high-confidence curated signaling maps. *Database* 2011:bar032. (2011).
